# Materials for Yeast Immobilization in Alcoholic Fermentation: Bridging Conventional Techniques and 3D Bioprinting

**DOI:** 10.3390/polym17233094

**Published:** 2025-11-21

**Authors:** Sara I. Brunet, Tamara Erceg, Ljiljana Janjušević, Slobodan Birgermajer, Mirjana Odanović, Vladimir Puškaš, Uroš Miljić

**Affiliations:** 1 BioSense Institute, University of Novi Sad, 21000 Novi Sad, Serbia; sara.brunet@biosense.rs (S.I.B.); ljiljana.janjusevic@biosense.rs (L.J.); b.sloba@biosense.rs (S.B.);; 2 Faculty of Technology Novi Sad, University of Novi Sad, 21000 Novi Sad, Serbia; tamara.erceg@uns.ac.rs (T.E.);

**Keywords:** yeast immobilization, alcoholic fermentation, biopolymers, 3D bioprinting, bioinks, hydrogels

## Abstract

Immobilization of yeast cells represents a significant advance in alcoholic fermentation. Compared to traditional methods that rely on the use of free cells, immobilized systems enable higher cell density, easier separation and reuse of biocatalysts, and improved fermentation control, all while maintaining cellular activity. The choice of immobilization material plays a key role in performance. Natural polymers such as alginate provide biocompatibility, but the main drawback is their insufficient mechanical strength. On the other hand, synthetic polymers offer greater durability but raise concerns regarding food safety and cost. Three-dimensional (3D) bioprinting is emerging as a promising solution, enabling the design of structural, customizable matrices with precise cell positioning and tunable physical properties. Traditional materials are undergoing reengineering as bioinks, while new synthetic and hybrid materials are being developed to overcome the limitations of conventional carriers. These innovations combine biocompatibility with mechanical stability and functional adaptability for industrial use. Although the application of 3D bioprinting to produce such carriers has shown promising progress, challenges remain in scalability, process integration, and long-term stability under industrial fermentation conditions. For these reasons, continued interdisciplinary research is necessary to further develop advanced techniques for immobilizing yeast cells for use in alcoholic fermentation.

## 1. Introduction

Yeast is fundamental to alcoholic beverage production because it enables the transformation of monosaccharides, such as glucose and fructose, into ethanol and carbon dioxide [1]. Among yeast species, *Saccharomyces cerevisiae* is predominantly used due to its distinct fermentative ability, high alcohol tolerance, and adaptability to various raw materials. It performs optimally at temperatures between 15 °C and 24 °C and plays a key role in, for example, ale beer production, where it produces complex mixtures of organic esters that are responsible for the characteristic fruity and spicy aromas [2]. In winemaking, carefully selected *S. cerevisiae* strains ensure efficient fermentation under acidic conditions and contribute to the expression of varietal aromas [3,4,5,6]. Furthermore, this species is also essential for the initial fermentation of raw materials used to produce spirits like whiskey, rum, and brandy [7]. In contrast, *Saccharomyces pastorianus* is used in the production of lager beer due to its ability to ferment at lower temperatures (7–15 °C), resulting in a cleaner and crisper flavor profile with reduced ester formation [2]. *Saccharomyces bayanus* is preferred in the production of higher alcohol wines and sparkling wines due to its higher alcohol tolerance (17–20%). Beyond these domesticated species, “wild” and non-*Saccharomyces* yeasts are increasingly utilized for their ability to enhance sensory complexity, especially in spontaneous and traditional fermentation [6,7]. Yeast metabolism is a major determinant of beverage quality as it directs the synthesis of volatile and non-volatile compounds, such as esters, higher alcohols, phenols, and diacetyl [4]. These metabolites shape key sensory attributes such as aroma, flavor, body, and mouthfeel. The selection of yeast strain, combined with precise control of fermentation parameters such as temperature, availability of nutrients, and oxygen exposure, decisively affects the chemical composition and individual character of the final product [2,4,6,7].

To further improve the efficiency and control of fermentation, modern biotechnological approaches have been developed. Among these, yeast cell immobilization stands out as a particularly important innovation. Immobilization of yeast cells has long been recognized as one of the most significant technological advancements in industrial fermentation, offering numerous operational and economic benefits compared to traditional free-cell systems. By physically trapping viable yeast cells at a certain location, immobilization makes it possible to achieve a high cell density, easy reuse biocatalysts, as well as better control of the fermentation process, without negative impacts on the metabolic activity of cells [8,9]. These advantages are in the form of improved fermentation kinetics, process stability, tolerance to stress conditions, and simpler downstream processing that make immobilized systems highly attractive to implement in industrial applications [10,11]. One of the most common methods of yeast immobilization is encapsulation in a porous matrix, which is most often applied using the technique of extrusion, in which the hydrogel mixture which contains yeast cells that are added drop by drop using a syringe. In this process, the drops are usually cross-linked upon contact with the solution, forming stable spherical beads containing encapsulated cells. This technique is widely used due to its simplicity, scalability, and ability to maintain high cell viability, making it suitable for various fermentation applications. The success of yeast immobilization methods, however, largely depends on the selection of a suitable support material. An ideal immobilization matrix would be a compromise between biocompatibility, mechanical strength, optimal porosity, chemical stability, and cost, with the condition that yeast cells remain alive and productive under industrial conditions. Furthermore, when food and beverage production is involved, regulatory compliance imposes an additional limitation that only food-grade, non-toxic materials may be utilized [9,12]. In this context, inert polymer materials have become increasingly prominent as immobilization carriers in alcoholic fermentation processes as they satisfy the above-mentioned requirements. Among the many materials explored for yeast immobilization, alginates have by far been the most used. Their gelation under mild conditions, high biocompatibility, and ability to form stable hydrogels make them an ideal candidate for entrapping yeast cells without influencing cell viability or activity [13]. Other natural and synthetic polymers, as well as inorganic materials have also been used, with each having some advantages but also specific limitations depending on the application [9,11,13].

In recent years, the field of cell immobilization has been significantly advanced by the development of three-dimensional (3D) bioprinting. This form of additive manufacturing enables precise layer-by-layer deposition of bioinks, typically composed of living cells, growth factors, and other biomolecules [14]. It also supports the creation of polymer-based scaffolds with embedded cells, offering precise control over their arrangement, porosity, and mechanical properties [15,16,17]. While originally focused on biomedical applications, 3D bioprinting has recently shown strong potential in biotechnology. It allows for the fabrication of complex structures that better mimic natural microenvironments, improved mass transfer, and enhanced fermentation efficiency. In the context of yeast immobilization, recent research has focused on developing printable bioinks with appropriate rheological properties while preserving cell viability and functionality. Despite its promise, the application of 3D bioprinting in yeast immobilization remains at an early stage. Traditional immobilization materials are being reevaluated and remodeled as bioinks, while new synthetic and hybrid materials are being created to overcome the limitations of traditional natural supports. While initial studies have reported promising results, scalability, process integration, and long-term stability under industrial fermentation conditions must be improved.

Unlike previous reviews that focused exclusively on conventional yeast immobilization systems (such as alginate, chitosan, or gelatin beads) or on the development of bioinks and 3D bioprinting in biomedical applications, this paper integrates both approaches. It conceptually positions yeast immobilization within the framework of engineered living materials (ELMs) and 3D bioprinting, presenting it not as a static cell entrapment procedure, but as a process for designing dynamic, living, and functional materials. A key new insight of this paper is that it views immobilization through the perspective of bioprinted architectures, taking into account the role of the selected polymer material, rheological/mechanical properties, printability, and structural control—parameters that have been largely neglected in previous reviews focused on fermentation processes.

## 2. Application of Polymers for Yeast Cell Immobilization in Alcoholic Fermentation

Polymers play a crucial role as the foundational materials for creating the scaffolds and matrices required for effective yeast immobilization due to their diverse chemical structures, tunable physical properties, and biocompatibility. These polymeric carriers provide a protective microenvironment for the yeast cells, facilitating their stability and reusability over multiple fermentation cycles. Polymers utilized for this purpose can be broadly classified based on their origin as natural or synthetic polymers. Natural polymers, originating from biological sources, are highly favored for yeast immobilization due to their inherent biocompatibility, biodegradability, and typically non-toxic nature. These materials, which include polysaccharides like alginate, chitosan, carrageenan, cellulose, and starch, alongside proteins such as gelatin and collagen, possess unique porous structures and chemical properties. These properties make them exceptionally well-suited for either entrapping or adsorbing yeast cells, providing a protective and functional matrix for fermentation processes. Synthetic polymers, engineered through precise chemical processes, offer significant benefits for yeast immobilization due to their customizable properties. Unlike natural polymers, these human-made materials allow for fine-tuning of their mechanical strength, chemical stability, and crucial pore size, which are vital for creating optimal environments for yeast cells. Polymers like polyvinyl alcohol (PVA), polyethyleneimine (PEI), polyurethanes, and various polyacrylates and polymethacrylates are widely used. Their versatility enables the design of highly robust and durable immobilization matrices, ensuring that yeast cells remain effective across a range of demanding fermentation conditions [10]. For instance, natural polymers are employed in applications where a close mimicry of the biological environment is required. In contrast, synthetic polymers are used because they provide precise control over molecular weight, structure, and functionalization, allowing for the development of materials with desired mechanical, chemical, and degradation properties [18]. The selection of an ideal polymer material for a particular application depends on a complex interplay of parameters, including biocompatibility, degradation kinetics, mechanical behavior, and interactions with cells or biological molecules. Among these various characteristics, the ability of polymers to form hydrogels is particularly critical, especially in applications focused on cell immobilization and advanced techniques such as 3D bioprinting. Hydrogels are particularly advantageous due to their three-dimensional, cross-linked polymer networks, which are highly hydrophilic. This structure allows them to absorb and retain large amounts of water while preserving mechanical stability, making them an effective medium for cell encapsulation [19]. This high-water content provides a soft, biocompatible environment that closely mimics physiological conditions, which are crucial for cell viability. In the context of yeast cell immobilization, particularly in 3D bioprinting, it is essential that the selected polymer be inert and capable of facilitating in situ cell entrapment without compromising cell viability. These crucial requirements are effectively met by various hydrogel systems. Acrylate-based hydrogels, formed through free-radical polymerization at room temperature, are suitable, as are hydrogels created via Schiff-base reactions or ionic interactions. However, the low viscosity of acrylate solutions often presents a challenge for their direct utilization in 3D bioprinting applications [20]. Given that many hydrogel systems rely on UV polymerization for crosslinking, careful selection of UV wavelengths is necessary to avoid biocidal effects on the encapsulated cells. Therefore, polymers capable of forming hydrogels under mild UV irradiation (such as polyacrylates, polymethacrylates, and modified biopolymers with double bonds) or via alternative crosslinking methods like Schiff-base formation and ionic interactions (e.g., alginate-based hydrogels) are particularly desirable for 3D bioprinting applications. Previous reviews about bioprinting such as those reported by Zoghi et al. [14] provide a detailed overview of basic bioprinting techniques, scaffold types, and bioinks, with an emphasis likely placed on mammalian systems given the usual orientation of such reviews towards tissue engineering. The review integrates the current knowledge on how hydrogel composition, crosslinking strategy (UV, ionic, or Schiff-base), and porosity regulation influence yeast survival, providing valuable insights into factors affecting bioethanol yield and cell concentration. Also, it integrates all the major immobilization approaches, with a particular emphasis on bioprinting technologies.

### 2.1. Natural Polymers/Macromolecules

Polysaccharides and proteins have been mainly used in the development of carriers for yeast cell immobilization employed in alcoholic fermentation. A summary of the characteristics of the materials used for yeast cell immobilization in alcoholic fermentation is presented in Table 1.

#### 2.1.1. Polysaccharides

Polysaccharides are a diverse and widely distributed group of natural macromolecules composed of monosaccharide monomers linked by glycosidic bonds. They have broad applicability. The commonly used polysaccharide biopolymers include cellulose, starch, chitosan, alginate, dextran, agarose, carrageenan, gellan gum, and xanthan gum. The chemical versatility of polysaccharides is one of their most important features. The fact that their structures contain many functional groups, such as hydroxyl, carboxyl, and amino groups, enables their chemical modification and cross-linking, which can be utilized for the creation of new materials with desired properties. This modifiability enables control of their solubility, mechanical strength, gelation capability, and interaction with other molecules [21,22,23]. The ability of these biopolymers to form hydrogels that respond to physiological stimuli makes them ideal for sustained drug release and for use in tissue engineering for developing scaffolds that mimic the extracellular matrix, thus supporting cell adhesion and tissue regeneration [23,24,25]. Among them, the most frequently used are chitosan, carrageenans, alginate, and gellan gum.

Chitosan, obtained by deacetylation of chitin, is a natural homogeneous linear polysaccharide primarily derived from the exoskeletons of crustaceans and insects, as well as from the cell walls of fungi and algae [26]. Soares et al. [27] reported that chitosan–alginate hybrid beads enhanced yeast tolerance to acetic acid concentrations up to 12 g L^−1^ during xylose fermentation with recombinant strains. The hybrid gels maintained cell viability and ethanol productivity under these inhibitory conditions, achieving a productivity of 1.13 g L^−1^ h^−1^, an ethanol yield equal to 75% of the theoretical value, and a 32% increase in xylose consumption. The biodegradability of chitosan and its food safety are particularly relevant for the production of alcoholic beverages. In the production of beer and sparkling wines, the encapsulation of yeast using a chitosan–alginate system facilitates simpler yeast recovery and significantly accelerates the disgorgement process. Additionally, these encapsulation methods yielded beverages with sensory profiles (aroma, flavor, and fullness) comparable to those produced by free yeast cells [28,29]. Naydenova et al. [28] further emphasized that chitosan-coated microcapsules enable process optimization by reducing primary fermentation time and increasing productivity through control of parameters such as initial extract concentration, temperature, and inoculum concentration. Despite the mentioned advantages, the use of chitosan-coated or combined beads has limitations. Mechanical instability, such as rupture after repeated or prolonged fermentation cycles, remains a problem, especially under agitation or under the acidic conditions typical of long-term beverage or ethanol fermentation [29,30]. In addition, the inclusion of chitosan may enhance mass transfer limitations within the immobilization matrix. The denser and less permeable structure of chitosan, although providing protection, simultaneously blocks the diffusion of substrates (e.g., sugar) and products (e.g., ethanol), leading to reduced fermentation rates and lower yields compared to fermentations with free suspended cells [28,30]. Duarte et al. [30] observed that the application of suspended yeast cells achieved an ethanol yield of 74–78% using glucose or sucrose, but chitosan–alginate beads yielded only 61–64% due to limited diffusion. During the production of sparkling wines, slow and incomplete fermentation was recorded when encapsulated unadapted yeast cells were applied to ethanol, which highlights the sensitivity of the process to the structure of the beads and cell physiology [29]. Another factor to consider is the sensitivity of chitosan to external conditions. Its behavior can be significantly conditioned by changes in pH and concentration of other dissolved substances, which can change its gelation ability, adsorption capacity, and mechanical properties in industrial fermentation conditions [27].

Carrageenans belong to the group of sulfated polysaccharides; they can be obtained by alkaline extraction of *Rhodophyceae* red algae. The most commonly used κ-carrageenan can cross-link and form hydrogels based on the physical crosslinking of sulfonate anions with K^+^ ions or polycationic polyelectrolytes, especially with chitosan [31,32]. To the best of our knowledge, recent and up-to-date research on the application of κ-carrageenan for yeast cell immobilization is relatively scarce, and most of the available findings come from earlier studies. Carrageenan, especially κ-carrageenan, has significant advantages as a matrix for yeast immobilization for fermentation purposes, but also serious limitations. One of the main advantages is that it can physically contain yeast cells, allowing concentrations ten times higher than those achieved in cell-free systems. For example, Wang and Hettwer [33] showed that κ-carrageenan beads can immobilize yeast cells at concentrations of 1 × 10^9^ cells mL^−1^, which is significantly higher than the 1 × 10^8^ cells mL^−1^ achieved in free-cell suspensions. This higher cell density enabled a more efficient conversion of nutrients into biomass, even under limited substrate conditions. It has also been shown that the addition of tricalcium phosphate crystals to the κ-carrageenan matrix further improves the performance of the system. The crystals stabilize the internal pH, improve cell viability, and increase the density and porosity of the gel. As a result, ethanol productivity can reach significantly higher levels. In industrial applications, particularly in brewing, κ-carrageenan gel beads have been used to enable continuous primary fermentation, greatly reducing the fermentation time from the usual 6–7 days to only 20 h. This improvement is attributed to the better mechanical strength and operational stability of the gel compared to alternatives such as alginate [12]. However, κ-carrageenan-based immobilization systems face a number of challenges. The high viscosity of the κ-carrageenan solution makes it difficult to form small, uniform beads. Larger beads are subject to limitations in mass transfer, leading to difficulties in substrate and product diffusion, and, in some cases, creating anaerobic microenvironments within the bead core even under aerobic conditions [33].

**Figure 1:**
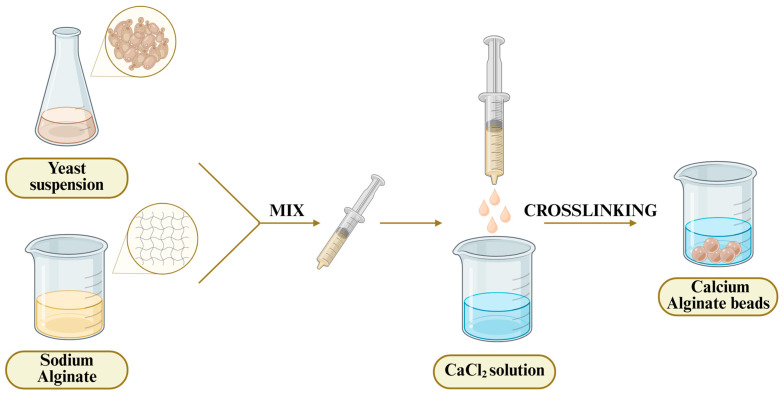
Conventional method of calcium alginate bead formation.

Sodium alginate is a water-soluble, linear polyanionic polysaccharide derived from marine brown algae. During the last decade, calcium–alginate beads have been among the most investigated and applied matrices for yeast immobilization in alcoholic fermentation (Figure 1). The beads can be obtained in mild conditions through replacement of sodium ions in guluronic parts with divalent Ca^2+^ cations [34]. Also, the other divalent cations such as Sr^2+^, Ba^2+^, etc., can be utilized in alginate bead formation.

There is a consensus among scientists about the advantages of using alginate due to its non-toxicity and biocompatibility, which allows for the immobilization of yeast without exposure to denaturation or stressful conditions. These properties ensure high cell viability and initial proliferation rates, as demonstrated in the work of Pajić-Lijaković et al. [35], where initial densities of up to 2.90 × 10^8^ CFU mL^−1^ were achieved in alginate beads. Damayanti et al. [36] also showed that 2% (*w*/*v*) sodium alginate resulted in beads with the highest sphericity, integrity, and textural homogeneity, capable of supporting repeated fermentation cycles, and yielding up to 88.13 g L^−1^ of ethanol in subsequent cycles. The industrial application of alginate also has practical advantages: it is cheap, widely available, and approved by regulatory bodies such as the International Organization of Viticulture and Wine (OIV), both as a clarification agent and as an acceptable yeast encapsulation medium in food and beverage fermentation [37]. Operationally, the immobilized yeast beads allow for easy separation from the final product, which significantly facilitates processes such as clarification, remuage, and disgorging in the production of sparkling wines where commercial Cremanti^®^ beads have shown exceptional stability during nine months of wine aging [37]. However, the literature also notes the limitations of using alginate as a matrix for yeast immobilization. A significant limitation of the use of calcium–alginate in vinification comes from the interaction of calcium ions with tartaric acid naturally present in wine. This reaction can lead to the formation of insoluble calcium–tartrate crystals, which result in unwanted sedimentation in the final product and potential impairment of visual quality [9,38]. Another disadvantage of using alginate is its increasing mechanical fragility over time. Repeated use, physicochemical changes in the medium, and high yeast metabolic activity lead to partial bead disintegration, increased porosity, and cell leakage. This reduces cell retention and process stability and can increase turbidity in the final product [35,36]. High cell densities further exacerbate these effects by accelerating structural degradation and reducing yeast vitality [36]. Damayanti et al. [36] state that immobilized yeasts quickly adapt to new substrates, but that the components of the medium, especially phenolic compounds, can have an unfavorable effect in combination with the alginate matrix. Also, the irregular and sometimes fragile internal structure of the beads affects the adsorption and diffusion profiles of nutrients and metabolic products, which not only affects the efficiency of fermentation, but also the purity and stability of the final product. Fernández-Fernández et al. [37] point out that wines fermented with immobilized yeast, although retaining most sensory and compositional characteristics, show lower concentrations of neutral polysaccharides and total proteins important for texture and fullness of flavor compared to wines fermented with free cells. This means that the porosity and permeability of the alginate matrix, although favorable for the diffusion of small molecules, limit the diffusion of larger macromolecules of yeast origin, which can affect the sensory and qualitative profile of the final product. For industrial applications, the problem of cell leakage from the beads has led researchers to further improve this system, for example, by including protective double-layer coatings for better cell retention, although such modifications may reduce the permeability of the matrix and limit the transfer of beneficial compounds. It can be concluded that alginate-based immobilization remains an attractive technology in yeast fermentation due to its mild encapsulation conditions, biocompatibility, low cost, regulatory support, and process simplification. However, its wide application is limited by the problems of mechanical fragility of the beads, cell leakage, incomplete diffusion of metabolites, and potential sensitivity to media components [35,36,37].

Another anionic polysaccharide with the ability to form gels with monovalent and divalent cations under mild conditions is gellan gum. Divalent ions, such as Ca^2+^ and Mg^2+^, are more effective promoters of gelation than monovalent ions such as Na^+^ and K^+^ so they form stronger gels [39]. Gellan gum comes in two forms: high-acyl and low-acyl gellan gum. High acyl gellan gum contains an acetate and glycerate group; one glycerate per repeating unit and one acetate for every two repeating units. These acyl groups can be removed by alkaline treatment of the fermentation broth at an elevated temperature. Cooling to approximately 65 °C produces soft, elastic hydrogels of high acyl gellan gum. Low acyl gellan gum is a deacylated product obtained by alkaline treatment and is commercially offered in the form of powdered gel particles. It differs significantly from the highly acyl form in gelation properties and mechanical strength. Upon cooling to 40 °C, it forms rigid, hard hydrogels that remain stable even at very low pHs [40]. Gellan gum is a matrix for the immobilization of yeast, with exceptional gelation properties and the ability to retain cells. Mântăluță et al. [41] reported that an efficient biocatalyst can be made using a precise combination of 0.4 g of gellan gum, 31 mL of calcium bentonite suspension, 12 mL of yeast suspension, and 40 mL of sterile distilled water. This mixture was extruded through a capillary in the form of balls, which were then stabilized in a calcium chloride solution. The gellan-gum beads showed excellent performance, particularly in retaining approximately 0.027 g of yeast biomass/g of beads. One of the significant advantages of this system is the ability to completely encapsulate the yeast cells within the gel matrix during fermentation, thus preventing cell leakage. As a result, the fermentation process produces a clear, crystalline wine without turbidity. Compared to conventional methods, yeast immobilization using gellan gum significantly improves fermentation efficiency as the entire process can be completed in 15 days, which is significantly shorter than traditional techniques. When using gellan-gum beads, remuage can be performed in just a few seconds by simply turning the bottle. However, key tasks for future research include optimizing the amount of beads per bottle, evaluating the long-term operational stability of the beads, and testing their reproducible use over multiple fermentation cycles. Gellan-gum-based matrices showed excellent reusability and long-term cell viability in repeated fermentation processes. In this context, Tincu et al. [42] proved that gellan-gum-based particles have high stability and preserved yeast cell viability above 88%, even after 84 h of exposure to an alcoholic medium. Each particle formulation (gellan or gellan/CMCNa) encapsulated 1.25 g of yeast, of which, 0.375 g were yeast cells (representing 30% of the total yeast mass). One of the main advantages of the system was the possibility of reuse because the particles retained uniform biocatalytic activity in up to nine fermentation cycles. Their stability was the result of the protection provided by the polymer matrix to the immobilized cells against the toxicity of ethanol and other environmental stressors. The study also compared pure gellan-gum beads with gellan/carboxymethylcellulose-sodium (CMCNa) composite beads prepared using magnesium as an ionic crosslinker. While pure gellan-gum particles showed superior fermentation efficiency after the fourth cycle, probably due to their higher porosity that facilitated substrate diffusion, gellan/CMCNa particles performed better in the first three cycles. This advantage of the cross-linked polymer composite is related to its denser and more stable network that allows greater penetration. However, the pure gellan beads had a slightly lower structural stability than the gellan/CMCNa composite, as indicated by the reduced transparency in the aqueous medium. Also, the pure gellan-gum formulations tended to release more yeast cells into the surrounding medium, indicating their poorer cell retention capability compared to composite matrices.

#### 2.1.2. Protein-Based Organic Polymers

Proteins are a significant class of natural macromolecules containing long chains of amino acids linked by peptide bonds. These macromolecules are fundamental to the structure and function of all living organisms and exhibit an extraordinary variety of properties owing to the diversity in amino acid composition, sequence, and organization. Their functional groups (amine, carboxyl, thiol, etc.) afford numerous chemical modification possibilities, enabling the creation of materials with customized physical and chemical properties [39,43]. Between them, the most frequently used is gelatin, which has the greatest potential for 3D bioprinting.

Gelatin is a natural protein that is produced by partial hydrolysis of collagen, which is present in the bones, skin, and connective tissues of animals, primarily pigs and cows. This biopolymer is known for its unique ability to form thermo-reversible gels. It easily dissolves in warm water, and upon cooling, it forms a gel, which is a property widely used in various processes of the food industry [44]. One of the key properties of gelatin is the firmness of the gel, which depends primarily on its amino acid composition and molecular weight. In the biomedical sector, chemically modified gelatin, known as GelMA (gelatin methacrylol or methacrylated gelatin), is used in wound dressings, tissue engineering scaffolds, and drug delivery systems thanks to its ability to form a gel under UV light, its compatibility with human tissues, and its ability to stimulate cell growth [45]. Several studies have shown that when gelatin is properly cross-linked using glutaraldehyde [46], chromium salts [47], or incorporated into a composite matrix with sodium alginate [48], carriers are created that allow for significantly higher initial fermentation rates and productivity compared to free yeast. For example, immobilized *S. cerevisiae* during glucose metabolism showed a significantly higher rate of conversion, almost twice as high compared to suspended cells [46]. In the works of Sungur et al. [47] and Devi and Nagamani [48], immobilized yeast retained their activity during many production cycles and during long-term storage, which confirms that properly prepared gelatin-based matrices can reduce the need for frequent renewal of the biocatalyst and thereby reduce process costs. In the case of immobilized yeasts, physiological changes were also recorded including accumulation of reserve polysaccharides, changes in macromolecular structures, disturbances in normal cell development, occurrence of polyploidy, and changes in the cell cycle. These phenomena are related to the immobilized state of the yeast and are in accordance with the wider scientific consensus on the negative impact of immobilization on the vitality and viability of the yeast [46]. The use of gelatin is limited due to its poor mechanical stability and strength, the instability of gelatin beads, and its susceptibility to microbial degradation. Therefore, gelatin achieves the best performance when it is part of a composite matrix or when the concentration of crosslinking agents (such as chromium) is carefully optimized [47,48].

**Table 1:** Advantages and disadvantages of natural polymers for yeast cell immobilization and fermentation.

Material	Product	Advantages	Disadvantages	Reference
Alginate and chitosan mixed before gelation	Ethanol	- Improved tolerance to inhibitors (e.g., acetic acid and other hydrolysate toxins) - Higher productivity and cell viability under stress - Enables cell reuse for more cycles - Higher xylose uptake rates under toxic conditions	- Preparation is more complex than alginate alone - Chitosan can slow substrate/product mass transfer, slightly reducing fermentation speed	[27]
Alginate beads with chitosan coating	Ethanol	- Repeated use for up to 8 cycles - Reduced risk of free-cell contamination/leakage - Slightly more robust than alginate alone	- Chitosan layer creates a barrier, slowing substrate/product diffusion and thus fermentation rates	[30]
Alginate beads with chitosan coating	Sparkling wine	- Greatly facilitates yeast removal (riddling/disgorging process) - Maintains desired sensory and foam characteristics - Non-toxic, food-grade, and aids cell physical retention	- Potential for capsule rupture, especially with gas evolution and energetic fermentation - Some mass transfer/diffusion limitations - Cell leakage is possible	[29]
Alginate beads with chitosan coating	Lager beer	- Enhanced fermentation productivity and cell stability - Facilitates continuous and batch operations - Improved operational control and cell reuse - Downstream processing simplified	- High biomass can lead to elevated metabolites that prolong maturation - Capsule breakdown is possible with rapid cell growth - Mass transfer effects need careful optimization	[28]
κ-carrageenan	Beer	- Higher mechanical strength - Enables continuous fermentation and reduced fermentation time (from 6–7 days to ~20 h)	- High viscosity can complicate small uniform bead formation - Larger-scale processes may experience greater bead size dispersion and increased diffusion limitations	[12]
κ-carrageenan with tricalcium phosphate	Ethanol	- Achieves much higher yeast cell concentrations than free-cell systems, yielding increased productivity - Physical retention allows for easy cell–product separation	- Mass transfer limitations for substrates and products, especially with larger bead sizes - pH control difficulties	[33]
Calcium alginate	/	- Good support for yeast immobilization - Maintains high cell viability - Enables rheological modeling of cell growth and matrix interaction	- Mechanical fragility and potential disintegration due to stresses generated by cell growth	[35]
Calcium alginate	Ethanol	- Enables repeated use in fermentation - High cell viability - Good mass transfer and colony growth inside beads	- Structural instability over extended/repeated use - Brittle defects at low alginate concentrations	[36]
Calcium alginate (Cremanti^®^)	Sparkling wine	- Non-toxic and OIV-approved for winemaking - Maintains yeast viability - Simplifies riddling/disgorging - Excellent cell retention, no cell leakage - Stable over 9 months of wine aging - No significant effect on wine parameters or effervescence	- Slightly reduced protein and neutral polysaccharide contents (may impact texture/mouthfeel) - Some large cell molecules may not diffuse well out of the bead - Minor aroma differences are possible	[37]
Gellan gum	Sparkling wine	- Effective yeast entrapment Faster fermentation (15 days) - Simple riddling	- Need for optimization of bead quantity - Long-term stability concerns	[41]
Low-acyl gellan gum beads and gellan/CMCNa composite beads, both crosslinked with Mg^2+^	Ethanol	- High yeast cell viability (>88% after 80 h in alcohol) - Stable biocatalytic activity over up to 9 cycles - High porosity in pure gellan beads supports efficient substrate diffusion, especially in later cycles - Composite beads (gellan/CMCNa) have greater mechanical/structural stability and reduced cell leakage in early cycles - Both are biocompatible and allow for easy recovery and reuse	- Pure gellan beads have lower mechanical stability over time and more cell leakage - Composite beads (with more CMCNa) may restrict substrate diffusion and yeast proliferation, reducing efficiency in later cycles - Both types can show a moderate decline in stability and efficiency after multiple uses	[42]
Glutaraldehyde-crosslinked gelatin	Ethanol	- Higher glucose uptake and ethanol productivity - The immobilized system maintained excellent viability throughout the experiment	- Reduced growth rate - Polyploidy - Diffusion limitations	[46]
Gelatin with chromium crosslinking	/	- High operational stability - Good enzyme activity yields - Reusability over 30 days	- Optimization needed for crosslinker concentration - Chromium salts can be toxic and may raise safety or regulatory concerns	[47]
Gelatin–alginate beads	Ethanol	- A combination of gelatin and alginate resulted in successful bead formation - Enhanced glucose uptake initially and supports high metabolic activity	- Gelatin alone could not form stable beads	[48]

### 2.2. Synthetic Polymers

The rapid development and application of synthetic polymers during the twentieth and twenty-first centuries have led to remarkable advances in the industry. Their durability and adaptable physical properties make them indispensable in sectors such as packaging, construction, electronics, textiles, energy, and automotive manufacturing. However, it is in the biomedical field that synthetic polymers have had a truly transformative impact. Innovations like hydrogels and advanced drug delivery systems have redefined the possibilities in medicine, offering solutions that were once out of reach [49]. What makes these materials particularly valuable is the precise control scientists have over their molecular structure and composition. This ability to adjust properties on the molecular level has rendered synthetic polymers vital materials in modern science and technology. Synthetic polymers are far more versatile than natural polymers, which originate from animals or plants. By carefully selecting different monomers and applying methods such as copolymerization and cross-linking, researchers can precisely adjust key properties like mechanical strength, chemical resistance, solubility, biodegradability, and biological compatibility [50,51].

Structural control can be achieved through cross-linking, where polymer chains are bonded into three-dimensional networks. Chemical cross-linking is the most prevalent technique for hydrogel construction, where a cross-linker of multiple functionalities, like N,N′-methylenebisacrylamide or ethylene glycol dimethacrylate, is added in the polymerization stage to develop covalent bonds between chains [52,53].

Alternatively, physical cross-linking is based on using non-covalent interactions such as hydrogen bonding, ionic pairs, or hydrophobic interactions to form reversible, physically interlocked networks. This process avoids the use of potentially toxic reagents, making the resulting products more biocompatible [50,51]. The major groups of synthetic polymers important for immobilization of yeast cells include polyethers, polyesters, poloxamers, polyacrylates and polyacrylamides, and polyvinyl derivatives, among others. Each group demonstrates specific characteristics that influence its application potential, particularly in advanced hydrogel systems, controlled drug release, diagnostic platforms, and tissue engineering solutions [44,53]. The main features of these materials are outlined in Table 2.

#### 2.2.1. Polyethers

Polyethers are a prominent class of synthetic polymers, where one of the most typical representatives is polyethylene glycol (PEG). PEG and PEG derivatives have gained popularity due to their hydrophilicity, flexibility, and superior biocompatibility. PEG is highly water-soluble and can be synthesized across a wide variety of molecular weights, allowing its properties to be fine-tuned for specific applications. PEG hydrogels are defined by their resistance to protein adsorption (“stealth” nature) and to design soft tissue-mimetic matrices [43,50,51].

#### 2.2.2. Poloxamers

Poloxamers are a family of synthetic triblock copolymers composed of blocks of polyethylene oxide (PEO) and polypropylene oxide (PPO); a well-known example is Pluronic F127. These polymers possess significant thermoresponsive behavior, for instance, being fluid at low temperatures and immediately gelling at body temperature. This property makes them attractive in injectable drug delivery systems and wound dressing applications because their sol–gel transition facilitates minimally invasive delivery and in situ gelation. Poloxamers are biocompatible and can be easily mixed with other polymers to achieve the desired mechanical and drug-release properties [50,51].

#### 2.2.3. Polyacrylates and Polyacrylamides

Polyacrylates and polyacrylamides are important classes of synthetic polymers that are obtained by polymerization of acrylic acid, acrylamide, and their various derivatives or copolymers. These hydrophilic polymers are favored for hydrogel research and biomedical investigations because of their extreme swelling behaviors and sensitivity to physical stimuli like pH and temperature [53]. Polyacrylates, such as poly(acrylic acid) (PAA), are characterized by carboxylic acid groups along their backbone. Upon ionization, these groups impart a high anionic charge density to the polymer. Crosslinking, typically achieved using N,N’-methylenebisacrylamide (MBAm), is crucial for forming hydrogels with exceptional water-absorbing and swelling capabilities [20]. The extent of these properties can be precisely regulated by adjusting the pH and ionic strength. Polyacrylate hydrogels are broadly applied for the controlled delivery of drugs, genes, and cells [19]. Poreda et al. [54] investigated the possibility of using polyacrylate hydrogels in the form of poly(potassium acrylate) (PAC) cross-linked with MBAm as carriers for the immobilization of *S. cerevisiae* in brewing. In this study, yeast cells were adsorbed on hydrated PAC gels. The starting yeast concentration in the fermentation liquid (wort) was 0.5 g L^−1^ (based on dry weight). The authors specifically examined the influence of this immobilizing material on the concentration of the main metal ions, such as calcium, magnesium, zinc, and manganese, in the fermentation medium. Their results showed that PAC was able to adsorb up to 50% of the cations present and significantly reduced the ion concentration in the solution. PAC hydrogels were also found to be strong, cost-effective, and satisfactory for efficient immobilization and reuse of yeast cells. However, research has shown that, in case of excessive intake, divalent cations can cause excessive cross-linking of the gel, reduce its ability to absorb water, and possibly reduce fermentative activity. Nevertheless, under the conditions tested, PAC-supported immobilization did not inhibit fermentation, and the yeast’s performance was at least as good as, if not better than, free yeast, even in repeated fermentation cycles, without added ions.

Research by Öztop et al. [55] was aimed at changing the ratio of acrylamide, sodium acrylate, and the type of cross-linker in order to immobilize yeast for ethanol production. The initial amount of yeast was 5 mg wet weight on 0.1 g of polymer. The authors optimized the swelling capacity and diffusion characteristics of the hydrogel to achieve maximum yeast immobilization and efficient metabolite transfer. It was found that a higher proportion of sodium acrylate promotes more intense nutrient uptake, a higher cell concentration, and higher ethanol productivity. MBAm was identified as the most effective crosslinking agent. The resulting hydrogels showed good operational stability, and the immobilized yeast could be reused in multiple fermentation cycles, with a gradual decrease in ethanol yield over time. The main drawback of these hydrogels is the risk of over-crosslinking, which leads to reduced porosity and poorer cell embedding, emphasizing the need to optimize the gel network to achieve optimal performance.

Polyacrylamide (PAAm) possesses amide instead of carboxyl groups, which gives it a neutral hydrophilic character and higher biocompatibility. PAAm hydrogels are often selected for their clarity, high tensile strength, and adjustable stiffness. Unlike polyacrylates, polyacrylamides are not pH sensitive, but through co-polymerization of acrylic acid or other monomers, can imbue them with desired responsiveness or improve physical characteristics such as porosity and stability [52,53]. One particularly fascinating derivative in this group is poly(N-isopropylacrylamide) (PNIPAM), which is well known for its thermoresponsivity. PNIPAM hydrogels have a dramatic phase transition at physiological temperatures (~32 °C): they are hydrophilic and swollen below this temperature but collapse and release water above it [49,52]. Mulko et al. [56] extended the application of polyacrylamide hydrogels using cryogelation, which obtained macroporous monolithic networks with strongly connected pores. Such a structure improved mass transfer and enabled high immobilization efficiencies ranging from 97.2 to 99.9% depending on the yeast loading. Cryogels have shown high mechanical stability, easy handling, and reusability, with preserved yeast vitality and high ethanol yields over multiple fermentation cycles. As a limiting factor, a drop in swelling capacity was observed at higher yeast loads, which can reduce the maximum cell concentration. Sun et al. [57] presented a different approach based on polymerization-induced phase separation, which formed macroporous polyacrylamide hydrogels with an open porous structure. The process was carried out in mild aqueous conditions with redox initiation using the yeast–potassium persulfate system. Almost complete efficiency of immobilization and uniform distribution of yeast in the matrix was achieved. The open-cell structure encouraged efficient mass transfer, which ensured high fermentation efficiency and stability in operation. After ten cycles of fermentation, the hydrogels retained about 86% of their initial performance, and also showed good stability during storage, especially in nutrient medium at low temperatures. However, challenges related to work precision and the risk of pore clogging due to yeast growth were highlighted. Zhaoxin and Fujimura [58] applied synthetic ionic hydrogels obtained by copolymerization of poly(ethylene glycol) dimethacrylate and 2-acrylamido-2-methylpropanesulfonic acid by gamma radiation-induced synthesis. In this way, hydrophilic matrices with controlled porous and mechanical properties were formed. Hydrogels enabled the binding and multiplication of yeast within their structure through the mechanisms of adhesion and proliferation. By increasing the glycol content, higher porosity and hydrophilicity were achieved, which significantly improved ethanol productivity compared to free cells. These results highlight the key role of carefully designed internal polymer architecture in achieving high fermentation efficiency.

#### 2.2.4. Poly (Vinyl Alcohol) (PVA) and Poly(Vinylpyrrolidone) (PVP)

Poly (vinyl alcohol) (PVA) and poly(vinylpyrrolidone) (PVP) are important vinyl-based synthetic polymers. PVA is noted for its biocompatibility, film-forming ability, and mechanical stability. PVA is applied in ophthalmology (e.g., eye drops and contact lens manufacturing), tissue engineering, and drug delivery. PVP, known for its solubility and innocuity, is traditionally applied to plasma expansion and drug delivery. Both polymers are able to form hydrogels with favorable swelling properties, and the properties can be optimized further by chemical or physical crosslinking procedures [49,50]. Martynenko et al. [59] demonstrated that immobilization of champagne yeast in PVA cryogels is an effective way to prevent cell escape during secondary fermentation, a critical parameter for maintaining clarity in sparkling wine production. They showed that properly prepared PVA cryogels retained yeast with minimal leakage while supporting sufficient fermentative activity, and that the matrix properties and yeast reactivation protocols could be tuned to optimize results. Nonetheless, the process requires careful preparation and control, which may be a barrier for broader commercial adoption. LentiKats technology, which utilizes PVA hydrogel in the form of lens-shaped particles for the immobilization of biological cells, was employed by Bezbradica et al. [60]. The authors reported successful immobilization that initially loaded the LentiKats carriers with an estimated concentration of 1 × 10^7^ cells mL^−1^. The PVA matrix proved conducive to cell proliferation, reaching high final cell loads of approximately 1 × 10^9^ cells mL^−1^ within the carriers. Immobilized yeast was shown to ferment well, reaching approximately 80% attenuation within two days and maintaining particle integrity and cell viability for thirty days of industrial operation. However, they did point out several material-related drawbacks: cells immobilized on a matrix exhibit a significantly longer lag phase and a lower maximum exponential growth rate compared to free cells. Moreover, during active fermentation, substantial cell leakage can occur due to pores formed in the matrix by CO_2_ production. Additionally, the technical requirements for preparing LentiKat further increase the operational complexity and costs. Together, these works suggest that, while PVA hydrogels like cryogels and LentiKat types are very beneficial for yeast immobilization in terms of stability, reusability, and retention with long-term stability during wine and beer fermentation and recombinant biocatalysis, the specific preparation procedures and sensitivity to process conditions are still major limitations to general acceptance.

**Table 2:** Summarized characteristics of synthetic organic materials for yeast immobilization.

Material	Fermentation Product	Advantages	Disadvantages	Reference
Poly(potassium acrylate) (PAC)	Beer	- Effective immobilization - Reusable - No calcium ion release	- Risk of over-crosslinking with excess divalent cations may slow or inhibit fermentation	[54]
Acrylamide–sodium acrylate copolymer hydrogels	Ethanol	- Adjustable swelling/porosity - High cell loading with higher sodium acrylate concentration - Increased ethanol yield - Reusable	- Over-crosslinking risks reduced porosity and cell embedding	[55]
Polyacrylamide cryogels	Ethanol	- High immobilization efficiency (97%) and large pore size - Facilitates mass transfer - Reusable - High cell viability	- Reduced swelling capacity with high yeast loading - Slower initial fermentation than free cells due to mass transfer limits	[56]
Macroporous poly(acrylamide) hydrogels	Ethanol	- Almost 100% immobilization efficiency enhanced mass transfer - High reuse capability - Uniform, in situ distribution of yeast cells	- Requires precise polymerization conditions to achieve optimal pore structure - Risk of pore blockage from yeast overgrowth	[57]
Poly(2-acrylamido-2-methylpropanesulfonic acid-co-poly(ethylene glycol) dimethacrylate) ionic hydrogel	Ethanol	- Adjustable porosity and mechanical properties - Enhanced ethanol productivity	- Complex preparation via gamma irradiation - Requires fine-tuning for optimal performance - Excessively large pores can reduce mechanical integrity and lead to cell leakage	[58]
PVA cryogels	Sparkling Wine	- Effective yeast retention - Minimizes cell escape - Produces organoleptic and chemical properties comparable to conventional methods	- Preparation complexity - Process control requirements	[59]
PVA (LentiKat particles)	Beer	- High cell loading - Good fermentation activity - Good mechanical stability	- Longer lag phase - Slower exponential growth - Eventual cell leakage - Preparation complexity	[60]

### 2.3. Regulatory Issues and Material Safety

Natural polymers have attained broad regulatory acceptance due to their established safety profiles and extensive history of use. For example, materials such as alginates (E 400–E 404), gellan gum (E 418), and gelatin are classified by the U.S. Food and Drug Administration (FDA) as Generally Recognized as Safe (GRAS) [61,62,63]. The European Food Safety Authority (EFSA) has conducted detailed re-evaluations of these substances, concluding that there is no need for a numerical Acceptable Daily Intake (ADI) for alginic acid and its salts and for gellan gum. Because of this regulatory status, these polymers may be employed in accordance with the Quantum Satis (QS) principle, meaning that they can be used in the minimum quantity necessary to achieve the desired technological effect, without a fixed numerical limitation [61,62]. This flexibility is particularly advantageous in large-scale manufacturing and fermentation processes, where precise dosage optimization is critical. Carrageenan (E 407/E 407a) has also achieved widespread acceptance and is recognized as GRAS by the FDA [64]. However, its current authorization by the EFSA is based on a provisional ADI of 75 mg kg^−1^ body weight per day [65]. Furthermore, it is important to note that natural polymers such as alginate, gelatin, and chitosan are approved for use in oenological applications by the International Organisation of Vine and Wine (OIV), in accordance with the International Code of Oenological Practices and the International Oenological Codex [66]. These approvals underscore their high industrial readiness level (IRL 8–9), signifying their suitability for immediate deployment in industrial processes.

In contrast to the wide regulatory acceptance of natural polymers, the commercial application of synthetic polymers is subject to much stricter control. This increased attention mainly comes from two key reasons: the possibility of degradation and leaching of residual, often toxic monomers, as well as the fact that most of these materials do not have clearly defined prior approval for use in contact with food on a larger scale. Therefore, the mechanical and chemical advantages of synthetic hydrogels are currently in the background compared to the demanding regulations that govern materials in contact with food. As a result, most of these materials are still considered unapproved for direct use in edible products. For instance, PVA is a widely used synthetic polymer; however, its approvals for food-related applications remain very limited. PVA itself is authorized as a specific food additive (E 1203) while its polyethylene glycol copolymers (E 1209) are strictly restricted to use as film-coating agents on solid food supplements [67,68]. This narrow scope of approval implies that large-scale industrial applications, such as yeast immobilization, would require extensive, case-specific safety evaluations and regulatory authorization for each individual substance intended for use as a food additive. Similarly, polyacrylates (e.g., PAA) and polyacrylamide are generally permitted only as processing aids in operations that do not result in detectable residues in the final product, with strict limits on residual monomer content. The U.S. FDA allows for the use of certain acrylate–acrylamide resins for enzyme immobilization, provided that the polymer contains no more than 0.05% (500 ppm) residual acrylamide monomer [69]. The potential leaching of acrylamide monomer, classified as a neurotoxic compound, represents a major regulatory obstacle, severely constraining the applicability of PAA-based systems in food-related processes. Complex photo-curable hydrogels, such as polyethylene glycol diacrylate (PEGDA), various Pluronic F127 derivatives (e.g., Pluronic F127 dimethacrylate and Pluronic F127-bisurethane), and photoinitiators such as lithium phenyl-2,4,6-trimethylbenzoylphosphinate (LAP), present additional regulatory challenges. The primary barrier to their use arises from the need to comply with specific migration limits (SMLs) and total migration limits for materials that come into contact with products of alcoholic fermentation. To demonstrate safety, comprehensive analytical testing is required to confirm that residual unreacted monomers (e.g., acrylate or methacrylate residues) and potentially leachable photoinitiator components remain below the legally prescribed thresholds [70,71]. Due to the large surface area and prolonged contact times characteristic of immobilization systems, meeting these safety requirements is a complex and costly process. In practical terms, this confines the industrial readiness level (IRL) of such advanced synthetic materials to stages 2–3, limiting their current use primarily to research and non-food applications.

## 3. Methods Used for Yeast Cell Immobilization

**Figure 2:**
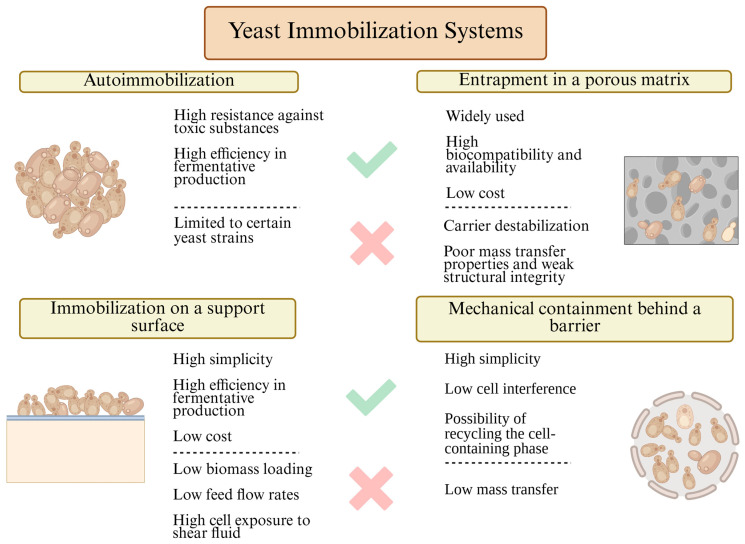
Advantages and disadvantages of immobilization techniques.

After the selection of suitable materials for immobilization, the choice of yeast immobilization method becomes crucial in determining the efficiency of fermentation bioprocesses [72]. Immobilization methods are generally classified into four main categories: mechanical entrapment behind a barrier, self-aggregation (auto-immobilization or flocculation), attachment to a solid support, and immobilization within a porous matrix. Comprehensive descriptions of these immobilization methods can be found in previous studies [8,9,13]. Here, we will briefly outline the main characteristics of each approach, emphasizing their relevance for yeast immobilization in fermentation processes (Figure 2).

*Mechanical containment behind a barrier* is a method where yeast cells are trapped within structures such as membrane filters, liquid–liquid interfaces, or microcapsules. This technique effectively preserves biomass and supports continuous operation but is usually beset with membrane fouling, increased operational complexity, and mass transfer constraints, especially under high-density fermentation conditions [9,13].

Another strategy utilizes the inherent auto-aggregation property of some strains of yeast, more accurately termed *auto-immobilization or flocculation*. These strains have an inherent propensity to spontaneously self-aggregate, form flocs, or develop biofilms through cell surface interactions and in response to specific environmental stimulants. The FLO gene family, particularly FLO1, FLO5, FLO8, and Lg-FLO1, plays a very significant role in regulating flocculation and adhesion processes in yeast and therefore becomes part of auto-immobilization. This impact has been used extensively in ancient winemaking and brewing processes since it is a cost-effective process and extremely simple to handle. However, broader use of auto-immobilization is still inhibited by its extreme reliance upon strain-specific properties and its severe sensitivity to changing process conditions, leading to notoriously poor reproducibility [11].

Next, *immobilization on a support surface* is based on yeast adhesion onto carriers made from materials such as cellulose, glass, ceramics, or organic substrates such as fruit pieces and wood chips [73]. It is a simple and extendible technique that offers a high surface area for immobilization. Cell attachment stability, however, can be influenced by mechanical agitation and movement in the fermentation medium, with tendencies for cell detachment and process inefficiency [9,11].

Lastly, *entrapment in a porous matrix*, such as alginate gels or synthetic polymers, is among the most applied methods for the entrapment of yeast cells in a semipermeable matrix. The configuration accommodates a high density of cells and reusability of the biomass for several cycles of fermentation [9,13].

**Figure 3:**
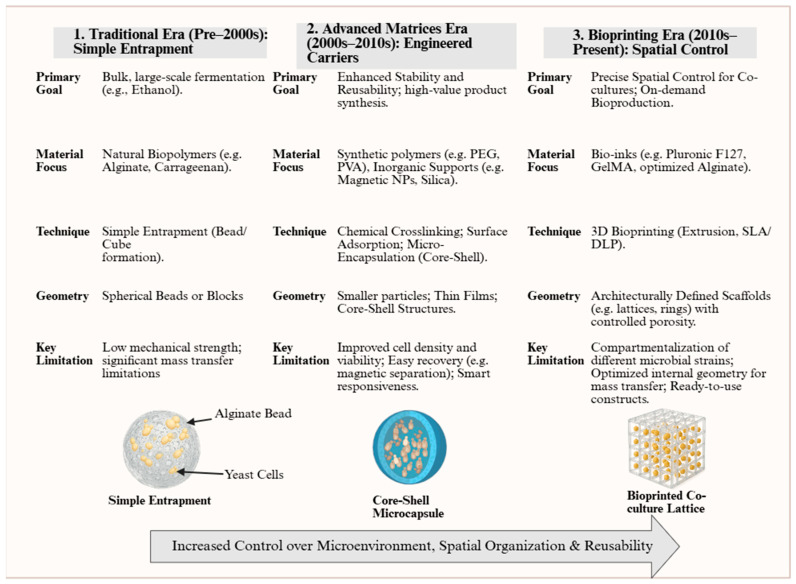
Evolution of yeast immobilization methods from traditional matrices to 3D bioprinting.

All these methods have played a vital role in advancing the process of yeast immobilization in fermentation processes. However, all of them suffer from inherent disadvantages, including limited control over the distribution of cells, mass transfer inefficiencies, mechanical instability, and scalability issues. All these disadvantages are more prominent when in search of increased productivity, process reproducibility, and economic viability in industrial applications today. Based on this, 3D bioprinting technology has developed in recent years, which could be applied to reduce the deficiencies described. The progression of yeast immobilization methods across successive periods, from early traditional matrices to more recent 3D bioprinting technology, is illustrated in Figure 3.

## 4. Three-Dimensional Bioprinting Techniques for Yeast Cell Immobilization

Unlike traditional 3D printing, which mainly relies on plastic or metal, 3D bioprinting aims to create structures with living cells in specially adapted material matrices, thus opening up possibilities that go beyond the scope of traditional manufacturing [74,75]. Such structures represent the basis of the so-called living materials, also known as engineering living materials (ELMs). These are composite systems in which living cells are embedded in a structural matrix, usually a hydrogel or a polymer scaffold, in order to provide the material with biological functions. The feature of living materials is that their functional properties, e.g., sensing, catalysis, self-healing, or environmental sensitivity, are derived from the physiological activity of the encapsulated cells. Microorganisms, e.g., bacteria, fungi, yeast, and microalgae, are usually employed as the living component due to their metabolic diversity, ease of genetic manipulation, and ability to grow in supportive matrices [15,16,75]. Three-Dimensional bioprinting uses several key techniques, each offering distinct advantages and facing specific limitations. As these methods have been thoroughly described in previous studies [75,76,77], an in-depth comparison is beyond the scope of this review; only a summary of their principal strengths and weaknesses will be provided. One such technique is *inkjet-based bioprinting*, which involves the usage of thermal or piezoelectric actuators to deposit small droplets of cell-containing bioink through microscale nozzles to build three-dimensional structures. This method is characterized by high speed, low cost, and high spatial resolution, which is suitable for precise cell placement and applications such as neural and skin tissue engineering. It is, nevertheless, limited to low viscosity bioinks and relatively thin constructs, as higher thickness or viscosity would be disruptive to both droplet formation and deposition [78,79].

*Extrusion-based bioprinting*, on the other hand, uses mechanical or pneumatic pressure to continuously extrude strands of highly viscous bioinks through a nozzle, which allows for the fabrication of large, robust, multilayered scaffolds. This process is compatible with high cell densities and a wide array of biomaterials and is therefore highly versatile for the production of bone, cartilage, and other mechanically demanding tissues. The main limitations are its relatively low printing resolution (features >100 μm) and potential cell damage from shear stress due to extrusion, necessitating careful optimization of the print parameters [74,80].

Another category, *laser-based bioprinting*, includes methods such as stereolithography (SLA) and laser-assisted bioprinting (LAB). SLA is based on the use of focused light to photopolymerize liquid bioinks layer by layer with high resolution and precision without exposing cells to mechanical forces. Developments in photoinitiators, such as a switch from UV- to visible-light-sensitive chemistries, have reduced cytotoxicity and improved the cell viability of SLA-printed tissues. LAB, wherein laser pulses are used to transfer bioink droplets from a donor surface to the build area, allows for extremely precise, nozzle-free cell placement and even single-cell per droplet placement. While cell viability has been reported to be very high with LAB, the process is complex, expensive, and currently less scalable than other approaches [80].

Even though the application of 3D bioprinting has been primarily focused on its application in biomedical research, it has demonstrated significant potential in application in the field of biotechnology in the past few years. This section provides an overview of the materials employed in the fabrication of such scaffolds using 3D bioprinting technologies. A substantial portion of research efforts has been directed towards the use of synthetic organic polymers, which offer superior tunability and mechanical properties compared to natural polymers. Natural polymers, on the other hand, have traditionally been explored for manually fabricated carriers, such as beads. However, their application in 3D bioprinting remains less prevalent, largely due to challenges related to their printability and structural stability under process conditions. An overview of the characteristics of materials that have been used in 3D bioprinting of yeast carriers is provided in Table 3.

In an effort to develop effective scaffolds for the immobilization of *S. cerevisiae*, natural organic polymers such as alginate, gelatin, and agar were used to fabricate structures via a free-form, motor-assisted microsyringe extrusion system. Each scaffold was inoculated with 1 × 10^6^ cells per well. This approach, as demonstrated by Aljohani et al. [81], resulted in scaffolds with high porosity (53–57%), a uniform pore distribution, and notable mechanical stability, including a maximum tensile strength of 31.21 MPa and a Young’s modulus of 0.83 GPa. Their hydrophilic nature, reflected in a contact angle of 65°, supports moisture retention, which enhances the long-term viability and metabolic activity of the immobilized yeast cells. The scaffolds’ biocompatibility was further confirmed through cell viability assays such as the Alamar Blue test, showing a high percentage of yeast cell viability (105% resazurin blue reduction), indicating that the materials are non-toxic and supportive of microbial growth. Although the combination of alginate, gelatin, and agar exhibits greater mechanical strength than each material individually, the elongation at break remained low (2.51%), indicating limited elasticity and a tendency toward brittleness under stress. The second limitation relates to the reproducibility and scalability of the bioprinting process. While 3D bioprinting has high precision, adjustment of printing parameters such as nozzle diameter, extrusion speed, and temperature may be complex and could require extensive adjustment to yield consistent scaffold quality and viability of cells. A study by Lopes et al. [82] introduced a new approach for alcoholic fermentation, with another natural organic polymer, alginate, used for the immobilization of cells. They constructed and incorporated a three-dimensional (3D)-printed millireactor, where *S. cerevisiae* cells were immobilized within a calcium–alginate hydrogel matrix. A culture medium was prepared with 1.2 g L^−1^ of dry and active yeast, while the ratio of sodium alginate to cell suspension was 1:1. With a redesigned 3D printer with both a fused filament fabrication system and a syringe module for the injection of hydrogel, they were able to automate and deposit cell-containing alginate precisely in targeted channels while building the reactor. This one-step fabrication process allowed them to create a functional microfluidic device that was immediately usable for continuous ethanol fermentation. Cell loss was negligible at low (1%) and high (6%) concentrations of alginate, although the higher concentration retained more cells. In particular, the ethanol yield was not significantly affected by alginate concentration, indicating that even at lower alginate levels, which decrease reagent costs, immobilization can remain effective. The immobilized system also eliminated the need for post-fermentation cell separation (e.g., centrifugation), simplifying downstream operations and reducing product loss. However, there were practical concerns of cell release at the lowest alginate concentration due to forming a less stiff matrix, and technical concerns with cell viability if the printing table was heated (which led them to use cold printing). Additionally, extremely thick hydrogel layers can lead to mass transfer limitations and substrate accessibility, which can impact the efficiency of fermentation if optimized appropriately.

**Figure 4:**
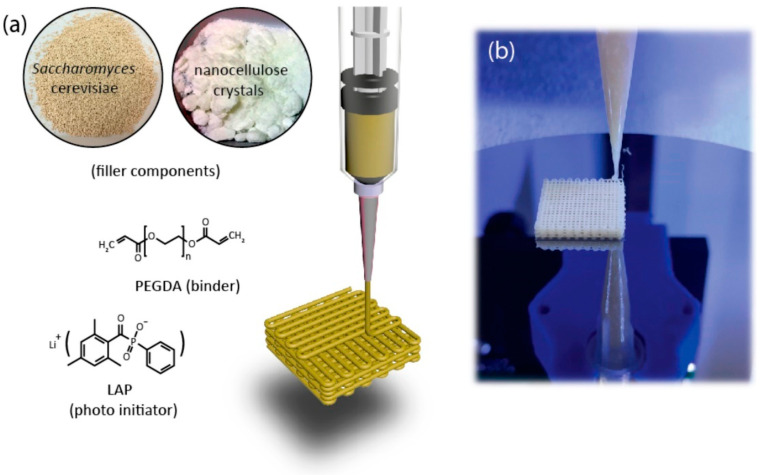
Schematic representation of the bioink components. (**a**) Scaffold obtained through 3D printing (**b**). Reproduced with permission from Ref. [16] (Copyright 2019, American Chemical Society).

The research of Qian et al. [16] was focused on using polyethylene glycol dimethacrylate (PEGDA), which belongs to the class of polyethers within synthetic polymers, specifically as a polyethylene glycol (PEG) derivative that has been chemically modified with methacrylate end groups. PEG derivative hydrogels are generally considered biocompatible, but not food grade. They developed a bioink composed of freeze-dried live baker’s yeast cells (*S. cerevisiae*) and nanocellulose as solid fillers, with PEGDA serving as the binder and lithium phenyl-2,4,6-trimethylbenzoylphosphinate (LAP) as the photoinitiator (Figure 4). Yeast granules and nanocellulose can be combined in nearly any ratio to create dual-filler bioinks, providing comprehensive control over ink rheology and cell density across a broad spectrum of formulations. Five representative inks were systematically formulated to demonstrate this control, where the yeast-to-nanocellulose weight ratio increased from 0 to 1, resulting in a cell loading range of 0% to 42.8 weight percent. Systematic studies determined the printable loading ranges for the single-filler bioinks and confirmed the roles of both components as viscosifiers. Nanocellulose inks were printable between 10 and 40 wt%. Increasing the nanocellulose loading from 15 wt% to 35 wt% resulted in a drastic increase in the plateau elastic modulus from 400 to 3500 Pa to over 60,000 Pa, alongside an order-of-magnitude increase in apparent viscosity. Similarly, pure yeast inks were printable between 30 and 50 wt%. Within this range, increasing the yeast concentration from 35 wt% to 50 wt% sharply raised the elastic modulus from 400 to 40,000 Pa.

**Figure 5:**
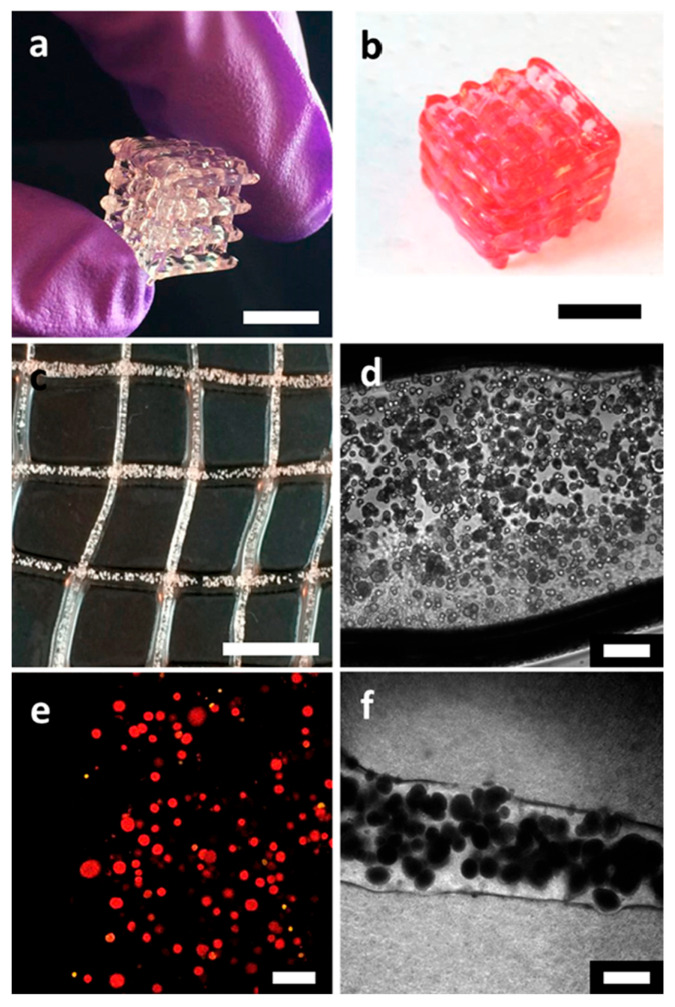
Images of 3D-printed cubes containing yeast cells (**a**) and (**b**). Images (**c**–**e**) show mCherry-expressing cell-laden 3D mesh structures on day 3: (**c**) a standard visual image, (**d**) an optical microscopy image, and (**e**) a confocal microscopy image, where Live/Dead staining with Sytox Green confirmed no significant cell death after 3 days of incubation in growth media. Image (**f**) presents an optical microscopy image on day 14. Reproduced with permission from Ref. [15] (Copyright 2018, American Chemical Society).

Using a direct ink writing (extrusion-based) 3D printer, the researchers created complex, self-supporting lattice structures capable of immobilizing yeast cells at exceptionally high densities with precise control over the cell distribution and scaffold architecture. The PEGDA hydrogel provided a safe and supportive matrix, maintaining yeast viability for months and preventing contamination. The 3D printed lattices produced approximately 16.1 g L^−1^ ethanol per 0.1 g ink within an hour, whereas traditional bulk hydrogel forms gave only 5.1 g L^−1^. The immobilized cells also maintained metabolic activity for longer periods and therefore, the scaffolds might be amenable to repeated or prolonged use. However, formulating the ink required fine-tuning since an excessive solid content made the ink too stiff for extrusion, while insufficient solids produced weak structures that were unable to hold their shape. Additionally, the hydrogel matrix tended to swell upon hydration, reducing effective cell density and altering the designed spatial distribution. There were also concerns regarding photoinitiator and light-curing effects on the viability of cells and some surface bacterial contamination that was found upon prolonged storage, which indicated potential limitations for prolonged stability in unsterile environments. There were scaling-up issues remaining as well: larger printed structures had lower ethanol production, possibly due to the aging of the cells or less mass transfer at the larger scale. Li et al. [83] developed an innovative approach for fabricating biocatalytic living materials using a specially designed, annealable granular hydrogel system suitable for 3D printing. Specifically, they combined thiolated sodium alginate (SA-SH) and hyperbranched poly(ethylene glycol) diacrylate (HB-PEGDA) to create hydrogel microparticles (HMPs). These HMPs encapsulated *S. cerevisiae* and, once combined together, served as a bioink for precise 3D structures using an extrusion-based 3D bioprinter. After printing, the constructs were further stabilized through calcium ion-induced binding, resulting in robust, porous scaffolds for supporting microbial activity. In terms of yeast immobilization, the materials and process offered several notable advantages. The microporous granular hydrogel structure enabled by SA-SH and HB-PEGDA provided an excellent environment for cell encapsulation, offering high protection from shear forces encountered during printing and preventing mechanical damage to the yeast. The crosslinking reaction was mild, which preserved cell viability and eliminated the risks associated with harsh photo initiators. The interconnected pores fostered nutrient and metabolite diffusion, thus supporting cell maintenance and proliferation within the scaffold. Also, there was significantly improved ethanol production compared to conventional bulk hydrogels, which was attributed to the macroporous structure of the printed lattices which enhanced mass transfer, creating optimal conditions for yeast metabolism. The immobilized yeast lattices offered reusability, maintaining high catalytic activity over multiple fermentation cycles and thus promoting sustainability and economic efficiency. However, this method was not without limitations. Achieving a uniform distribution of yeast among the beads was inherently difficult due to the random nature of encapsulation; some HMPs remained empty, and optimization was necessary to reduce this variation. Over long-term use, there was also the issue of cell leakage resulting in turbidity of the fermentation media, indicating a degree of lost containment. While not a major issue in the short term, this could impact process stability and would need to be addressed for large-scale or continuous operations. Additionally, the eventual overgrowth of yeast within the scaffold could, over time, alter the structural and functional integrity of the printed material. Both Saha et al. [15] and Žunar et al. [84] have investigated the use of Pluronic F127-based hydrogels from the synthetic poloxamer group and specifically modified with dimethacrylate (F127-DMA) as platforms for yeast immobilization in engineered living materials. Although their methodologies and research focuses differ, they offer complementary insights into the properties of these materials. Both groups prepared the hydrogels using a similar process: an F127-DMA solution was combined with a yeast suspension and the photoinitiator 2-hydroxymethyl propiophenone, followed by UV irradiation to induce crosslinking. Žunar et al. [84] used genetically encoded biosensors and confocal microscopy to assess cell–scaffold interactions at the single-cell resolution, focusing on the metabolic state, growth phase, and microcolony formation of *S. cerevisiae* within cross-linked F127-DMA hydrogels. They discover notable advantages, including a high viability of the cells, the capacity to promote (micro)aerobic environments, and the potential to incorporate functional whole-cell biosensors while avoiding contamination. Yet, one of the disadvantages highlighted was the high nutrient gradient in thick hydrogels with consequent non-uniform cell growth and larger and denser microcolonies at the periphery where there is greater diffusion of nutrients, and smaller, potentially less active populations within the interior. This excludes homogeneity and may limit scalability of bioprocesses. Alternatively, Saha et al. [15] dealt with the challenge from the process optimization and material science point of view by developing a multi-stimuli-responsive, F127-DMA hydrogel ink that accommodates yeast and is directly 3D printable (Figure 5). They demonstrated that the system enables the reproducible production of elastic and durable 3D lattices. A major advantage of this approach is the hydrogel’s reversible sol–gel transition at low temperatures, which allows for homogeneous yeast loading while the photoinitiated cross-linking stabilizes the cells. Crucially for extrusion, the hydrogel showed ideal shear-thinning behavior—viscosity decreased with increasing shear rate, which is characteristic of a non-Newtonian fluid. This shear thinning was fully reversible, which is essential for the extruded filament to maintain its shape immediately after exiting the nozzle. Furthermore, the hydrogel showed minimal mechanical hysteresis across strain cycles, making it highly desirable for extrusion fabrication. Photochemical crosslinking was employed, confirmed by photorheometry, which showed a rapidly increasing elastic modulus that increased 9-fold after just 60 s of irradiation, indicating a successful, fast conversion from a physically to a chemically crosslinked network. For fermentation applications, they reported several benefits: the material provides longer yeast viability and catalytic activity, efficient glucose-to-ethanol conversion for at least two weeks (∼90% yield), and design versatility for tailored bioreactor geometries. The potential disadvantages mentioned included surface cell escape, the need for photoinitiator and UV illumination, and restricted mass transfer or substrate diffusion due to active populations, affecting the overall bioprocess effectiveness.

Current research on the application of Pluronic F127-bisurethane methacrylate (F127-BUM) hydrogels in yeast immobilization has been thoroughly explored in several studies [85,86,87]. Johnston et al. [85] fabricated both mono-culture and co-culture scaffolds using F127-BUM hydrogels through extrusion-based 3D printing with good mechanical stability, high processability and long-term cell viability. The printed hydrogel lattices containing *S. cerevisiae* sustained high levels of ethanol production over the course of a full year of repeated batch fermentations. Their research also uncovered the superior preservation abilities of hydrogel-based bioreactors, such as successful lyophilization and rehydration cycles for on-demand and repeated bioproduction. However, they did mention frequent problems such as small cell leakage with extended usage and further optimization was needed to ensure the complete exclusion of foreign cells, particularly in dynamic or long-term systems. To add to this, Johnston et al. [86] focused on the physicochemical attributes of F127-BUM, emphasizing the use of temperature-sensitive sol–gel transitions and photocrosslinking for encapsulating diverse cell types and designing multi-kingdom constructs. They mentioned high cell viability and clear-cut compartmentalization as major advantages but also stated that barriers to diffusion in thicker hydrogels are capable of introducing oxygen and nutrient gradients, which inhibit colony growth and function in central regions. Butelmann et al. [87] demonstrated that F127-BUM hydrogels establish a microaerobic environment due to highly impaired oxygen diffusion, which forces immobilized yeast towards fermentative metabolism. Due to this fact, they conducted experiments with beer and ethanol production using yeast immobilized in these hydrogels. Both systems showed that yeast inside these hydrogels produced more ethanol compared to traditional suspension cultures: specifically, a 3.7% higher alcohol content in the beer that was produce using immobilize yeast after 14 days of fermentation and a 13.6% higher ethanol yield in batch fermentation using glucose as a carbon source. Therefore, while F127-BUM hydrogels possess higher structural stability, regulatable cell targeting, and reuse compatibility for fermentations, the extension of their applications to aerobic bioproduction requires additional material and process development aimed at improved oxygen and nutrient supplies.

**Table 3:** Materials used in 3D bioprinting production of yeast carriers.

Material	Fermentation Product	Advantages	Disadvantages	Reference
Alginate, Gelatin, and Agar	Ethanol	- High porosity and homogeneous pore distribution - Mechanical stability - High yeast viability - Non-toxic	- Brittle and low elasticity - Complex bioprinting parameter adjustments - Scalability and reproducibility challenges	[81]
Calcium–Alginate Hydrogel	Ethanol	- Biocompatible and nontoxic - Minimal cell loss - Homogeneous immobilization - Reusable	- Cell leakage at low alginate concentrations - Mass transfer limitations in thick hydrogels - Requires careful adjustment of printing and operational parameters	[82]
PEGDA and Nanocellulose	Ethanol	- High-density cell immobilization - Prolonged viability - Contamination resistance - High ethanol yield - Reusability	- Ink formulation sensitivity - Hydrogel swelling reduces cell density - Photoinitiator toxicity concerns - Scaling up issues	[16]
SA-SH and HB-PEGDA	Ethanol	- Gentle crosslinking preserves viability - Microporous structure enhances - Mass transfer - High ethanol production - Reusable	- Uneven yeast distribution - Cell leakage over time - Scaffold structural integrity affected by overgrowth	[83]
F127-DMA Hydrogel	Ethanol	- Uniform cell encapsulation - Robust mechanical properties - Efficient glucose-to-ethanol conversion - Design flexibility	- Some cell escape at surface - Photoinitiator/UV may pose toxicity risk to cells - Mass transfer limitations in thicker structures	[15]
F127-DMA Hydrogel	Ethanol	- High cell viability - Supports microcolony formation - Contamination avoidance	- Central zones suffer from nutrient/oxygen limitations - Non-uniform cell growth - Scalability limitations	[84]
F127-BUM Hydrogel	Ethanol	- Enables long-term, repeated, on-demand bioproduction - Stable high yields in repeated batches - Long-term viability	- Cell leakage during long-term use - Susceptibility to medium/pH/ionic strength - Diffusion barriers in thick constructs	[85]
F127-BUM Hydrogel	Ethanol	- High cell viability, temperature-sensitive sol–gel transition, and good compartmentalization	- Diffusion gradients limit central cell growth in thicker hydrogels - Surface adherence by foreign microbes	[86]
F127-BUM Hydrogel	Ethanol, Beer	- Enables 3D printing of stable, cell-laden structures - Enhanced ethanol yield (+3.7% in beer) - Robust, reusable, and easy cell recovery	- Higher material and fabrication costs - Some cell leakage after prolonged use - Slower substrate and product diffusion that extends fermentation time - Variations in beer color and taste that require further optimization	[87]

### 4.1. Challenges and Future Prospects of 3D-Bioprinted Materials for Yeast Immobilization

#### 4.1.1. Nutrient Transport Limitations

One of the main challenges in forming 3D-bioprinted scaffolds for immobilizing yeast cells is addressing the problem of mass transfer within the hydrogels, which hinders the biological stability of the cells. Nutrient diffusion limitations, particularly for glucose and other essential substrates, create pronounced spatial heterogeneity, with the outer regions supporting active growth, but the central regions characterized by nutrient deprivation and metabolic stagnation [84,86]. A study by Johnston et al. [86] concluded that printing structures with features thicker than 500 µm likely does not provide adequate diffusion of nutrients to the cells at the core of the hydrogel, resulting in colonies located near the periphery of hydrogels. Such diffusion gradients create physiologically distinct microenvironments, hindering the possibility of homogeneous activity and scalability of living materials.

To overcome this barrier, future research must integrate architectural, material, and biological innovations. Advanced multi-material 3D printing techniques can embed microfluidic channels or perfusion networks directly into hydrogel constructs, transforming mass transfer from passive diffusion to active convection and maintaining a homogeneous distribution of nutrients [88]. Solutions also involve designing lattice structures that maximize surface-area-to-volume ratios and using multi-level structures to enhance scaffold porosity and improve mass transfer [15,17,82]. Also, using genetically encoded biosensors inside the encapsulated yeast, researchers can track metabolic signals such as ATP levels and cytosolic pH in real time and at the single-cell resolution. This information can help them adjust the 3D scaffold design and bioink formula to reduce the strong nutrient gradients that currently limit cell growth [84].

#### 4.1.2. Long-Term Stability and Cell Outgrowth

When it comes to the long-term application of the obtained immobilization systems, a practical limitation has been observed for cell growth. Yeast cells proliferate within the hydrogel matrices over time, where they can migrate outside them and enter the final fermentation product [15]. Also, the mechanical properties of hydrogel matrices change during a longer period of cultivation. As a result, there is progressive degradation of polymer networks, an accumulation of metabolic by-products, and progressive densification of cell populations, which changes the structural integrity of the structure and characteristics of gas/nutrient transport [15,89]. This introduces unpredictability into the long-term performance of bioprocesses.

One possible solution to this problem, which allows for the control of cell growth and a reduction in mechanical degradation, is the application of a lyophilization process (freeze-drying) [85]. Research by Johnston et al. [85] showed that the mechanical properties of hydrogels remain stable after lyophilization and rehydration whereas Young’s moduli before and after conservation were identical. Moreover, scanning electron microscopy (SEM) imaging indicated minimal change in microstructure. An additional benefit of using this method is that it was observed that the overall rate of cell leakage from the gel was reduced after lyophilization and rehydration compared to pre-lyophilization conditions. Furthermore, yeast-loaded hydrogels maintained 100% production efficiency after rehydration even after 3 months of storage at room temperature. Another suggestion could be that future bioprinter design should include integrated sensors and monitoring systems capable of non-invasively detecting cell viability and population density within different construct regions, the accumulation of metabolic byproducts, and changes in mechanical properties [88,90]. Such monitoring systems can provide early warnings of construct degradation and the initiation of cell growth, enabling predictive maintenance to prevent fermentation failure.

#### 4.1.3. Scaling Challenges

One of the biggest obstacles when it comes to applying 3D bioprinting for immobilization of yeast cells is the transition from the laboratory to the industrial scale. As Pu et al. [75] mentioned, the large-scale use of 3D bioprinting technology lacks optimization, whereas most applications today use small batch or customized production. In contrast, industrial fermentation relies on production at much larger scales, typically hundreds to thousands of liters for commercial production. Scaling up production by simply multiplying small, independent 3D-printed biostructures is an inefficient and unsustainable strategy because it multiplies the costs, labor, and maintenance rather than integrating them into a single, automated, high-throughput system that is necessary for industrial efficiency. Erkan Ünsal et al. [17] showed that 3D-printed spheres operating through repeated batch cycles show a decline in productivity after the seventh cycle, indicating that even well-functioning laboratory-scale systems lose efficiency over time. For biotechnological applications, such limitations present major challenges as large-scale production relies on continuous or semi-continuous processes. It must be pointed out that the problem lies in fact that 3D bioprinting technology was primarily developed for the field of tissue engineering, where small volumes and a high degree of customization can be of value. Considering the different 3D bioprinting techniques, extrusion-based bioprinting, which is based on sequential layer-by-layer deposition, is the most labile technique. Precisely because of this, the technique becomes very time-consuming when producing large constructs. The process is therefore labor-intensive and rather slow, and therefore poorly suited to the requirements of industrial bioprocessing, where consistency, speed, and cost-effectiveness are required [91,92].

To address this challenge, researchers should also focus on systematically redesigning bioprinting approaches for implementation in biotechnological manufacturing. This shift would move development beyond the constraints of tissue-engineered systems and toward platforms specifically optimized for industrial production. In such contexts, bioprinters should prioritize printing speed, scalability, and throughput over resolution or biological adaptation [80]. Secondly, a promising direction involves volumetric printing technologies, which enable the simultaneous fabrication of large-scale living materials and entire constructs through holographic or interference-patterned light exposure [75]. These methods could reduce fabrication times from hours to seconds, eliminating the time barriers of extrusion-based bioprinting. Although current volumetric techniques are designed for sensitive mammalian cells, the robustness of yeast cells could allow more aggressive processing conditions to be used, potentially enabling industrial-scale bioprinting with high throughput and reproducibility [75,93]. Achieving industrial scalability also requires standardization in all aspects of the process. Due to this, standardized construct geometries, universal bioinks, integrable G-code files, common metrics for porosity, mechanical properties, and cell viability should be defined and verified through international standards organizations [75,94]. Finally, scalability will require real-time monitoring, predictive analytics, and data-driven optimization to maintain quality and consistency. Artificial intelligence and machine learning will be essential in identifying optimal printing parameters, material formulations, and improving quality assurance. These analytical tools would make workflows more efficient, reduce costs, and maintain consistent large-scale production [80,90,94].

#### 4.1.4. Cost Implications

Another significant barrier is the expense involved in applying 3D bioprinting technology. In general, commercially available bioprinters vary considerably in price, from basic systems priced around USD 5000 to advanced platforms that can reach prices up to USD 300,000 [95]. In addition to the price of the printer itself, the accompanying infrastructure should be considered, such as equipment for media preparation and incubation, sterilization systems, and analytical tools for monitored productivity, which all add to the capital investment. On the other hand, Pu et al. [75] highlighted that although technological advances will gradually reduce equipment costs, highly efficient and environmentally friendly materials will remain relatively expensive. Natural polymers are affordable and sustainable, with alginate being widely used due to its low cost and simple gelation. Food-grade sodium alginate is readily available in bulk for about USD 5–10 per kilogram. However, natural materials can suffer from batch variability and low mechanical strength, leading to shorter lifetimes and frequent replacement in high-shear bioreactors [21]. In contrast, synthetic polymers offer high mechanical, chemical, and thermal stability with precise structural control, making them more durable and reliable and often offsetting their higher production costs through reduced replacement and maintenance needs [75]. For example, PEGDA costs approximately USD 240 per kg, although prices may vary depending on purity, molecular weight, and purchase volume. In comparison, the commonly used UV photoinitiator Irgacure 2959 can be found at about USD 150 for 5 g, making it a considerably more expensive component on a per-mass basis.

Despite these challenges, there are several options that could enable industrial-scale production. First, the development of sustainable, renewable bioinks could significantly reduce material costs while improving environmental appeal. Krujatz et al. [90] suggest that new biomaterial inks can be developed from bio-resins and plant fibers due to their beneficial properties. Additionally, organic residues and living microorganisms such as fungi or algae, with their diverse morphologies, provide innovative options for creating matrix-forming compounds. Such bio-based alternatives could dramatically reduce per-unit material costs while aligning with circular economy principles. Second, advances in multi-material printing technologies offer potential for optimizing cost–benefit trade-offs. Rather than printing entire constructs with expensive, high-performance bioinks, hybrid approaches could use inexpensive structural materials (like polycaprolactone or PLA) for load-bearing frameworks, reserving costly bioinks only for cell-containing regions. This strategy, while requiring technological sophistication, could reduce material costs without compromising biological function [74].

## 5. Conclusions

The choice of materials represents the basis of successful immobilization of yeast in alcoholic fermentation because it directly determines the efficiency of the process, its stability, and the possibility of wider application in modern biotechnological procedures.

Traditionally, natural polymers, primarily alginates, are the most widely used due to their favorable properties such as biocompatibility, non-toxicity and easy handling, and the additional advantage is that they are approved for use in the food industry. Their low price and ability to provide a mild and protective microenvironment for yeast make them particularly suitable for processes such as the production of alcoholic beverages or fermentation to obtain bioethanol. However, their lower mechanical resistance, cell leakage, and gradual disintegration during repeated use remain key drawbacks that limit their long-term application.

Synthetic polymers, on the other hand, bring certain advantages that natural materials can hardly provide. They are characterized by greater structural strength, better durability, and resistance to mechanical and chemical stress so they are especially valuable in situations where the priority is to reuse the carriers and maintain stability over a long period of time. However, these advantages are accompanied by significant limitations: higher prices, weaker biocompatibility, and food safety concerns.

Compared to traditional immobilization methods such as bead formation, 3D bioprinting represents an advance in quality by enabling the printing of immobilization matrices with controllable design, structural precision, and defined cell arrangements. Modern bioprinters equipped with multiple tools and modular systems allow for the use of a wide range of materials and printing strategies, expanding the possibilities for design and process optimization. With this technology, it is possible to use new bioinks and composite materials with adjustable porosity and high mechanical strength. Although still in the early stages of yeast immobilization, 3D bioprinting has clear advantages in structural control, reproducibility, and functionalization. Since the main focus of the research is on the formation of carriers for ethanol production, it would be useful to examine their application in the production of beverages such as wine, beer, and cider in more detail. In these cases, immobilized yeast could contribute to easier process optimization and enable more efficient biotechnological workflows. However, major challenges remain regarding scalability, regulatory approval, and integration into existing industrial processes. Further development of this field will rely on the close integration of materials science, bioprinting technology, bioprocess engineering, and systems biology.

## Data Availability

No new data were created or analyzed in this study.

## Abbreviations

The following abbreviations are used in this manuscript:

PVA	polyvinyl alcohol
CMC	carboxymethylcellulose
PEO	polyethylene oxide
PPO	polypropylene oxide
PAA	poly(acrylic acid)
MBAm	N,N’-methylenebisacrylamide
PAC	poly(potassium acrylate)
PAAm	polyacrylamide
PNIPAM	poly(N-isopropylacrylamide)
PVP	poly(vinylpyrrolidone)
FDA	Food and Drug Administration
GRAS	Generally Recognized as Safe
EFSA	European Food Safety Authority
ADI	Acceptable Daily Intake
QS	Quantum Satis principle
OIV	International Organisation of Vine and Wine
SMLs	specific migration limits
IRL	industrial readiness level
ELMs	engineered living materials
SLA	stereolithography
LAB	laser-assisted bioprinting
PEGDA	polyethylene glycol dimethacrylate
HMPs	hydrogel microparticles
PEG	polyethylene glycol
LAP	lithium phenyl-2,4,6-trimethylbenzoylphosphinate
SA-SH	thiolated sodium alginate
HB-PEGDA	hyperbranched poly(ethylene glycol) diacrylate
F127-DMA	Pluronic F127 dimethacrylate
F127-BUM	Pluronic F127-bisurethane methacrylate
